# Network Diffusion Approach to Predict LncRNA Disease Associations Using Multi-Type Biological Networks: LION

**DOI:** 10.3389/fphys.2019.00888

**Published:** 2019-07-16

**Authors:** Marissa Sumathipala, Enrico Maiorino, Scott T. Weiss, Amitabh Sharma

**Affiliations:** ^1^Channing Division of Network Medicine, Department of Medicine, Brigham and Women’s Hospital, Harvard Medical School, Boston, MA, United States; ^2^Harvard College, Cambridge, MA, United States; ^3^Department of Medicine, Harvard Medical School, Boston, MA, United States; ^4^Center for Interdisciplinary Cardiovascular Sciences, Cardiovascular Division, Brigham and Women’s Hospital, Harvard Medical School, Boston, MA, United States

**Keywords:** lncRNA, network medicine, interactome, network diffusion, disease, protein–protein interactions, disease network

## Abstract

Recently, long-non-coding RNAs (lncRNAs) have attracted attention because of their emerging role in many important biological mechanisms. The accumulating evidence indicates that the dysregulation of lncRNAs is associated with complex diseases. However, only a few lncRNA-disease associations have been experimentally validated and therefore, predicting potential lncRNAs that are associated with diseases become an important task. Current computational approaches often use known lncRNA-disease associations to predict potential lncRNA-disease links. In this work, we exploited the topology of multi-level networks to propose the **L**ncRNA rank**I**ng by Netw**O**rk Diffusio**N** (LION) approach to identify lncRNA-disease associations. The multi-level complex network consisted of lncRNA-protein, protein–protein interactions, and protein-disease associations. We applied the network diffusion algorithm of LION to predict the lncRNA-disease associations within the multi-level network. LION achieved an AUC value of 96.8% for cardiovascular diseases, 91.9% for cancer, and 90.2% for neurological diseases by using experimentally verified lncRNAs associated with diseases. Furthermore, compared to a similar approach (TPGLDA), LION performed better for cardiovascular diseases and cancer. Given the versatile role played by lncRNAs in different biological mechanisms that are perturbed in diseases, LION’s accurate prediction of lncRNA-disease associations helps in ranking lncRNAs that could function as potential biomarkers and potential drug targets.

## Introduction

Non-coding RNAs can be classified broadly in two types: small non-coding RNAs and long non-coding (lnc) RNAs that are more than 200 nucleotides (Kapranov et al., 2007; Kung et al., 2013). LncRNAs are discrete transcription units located in sequence space, which do not overlap protein coding genes (Kung et al., 2013). Recently, lncRNAs have received widespread attention due to their diverse roles in biological regulation, developmental processes, and diseases (Mercer et al., 2009; Orom et al., 2010; Moran et al., 2012; Sun and Kraus, 2015; Ulitsky, 2016). With a wide array of regulatory functions in epigenetic, transcriptional, post-transcriptional regulation including histone modification, DNA methylation, and transcriptional co-regulation, it is not surprising that the dysregulation of lncRNAs have been reported in many diseases (Liu et al., 2013; Shi et al., 2013; Chakravarty et al., 2014; Kataoka and Wang, 2014; Ma et al., 2016). Furthermore, increasing evidence suggests that the regulatory role of lncRNAs in biological processes often involves interactions with proteins (Ferre et al., 2016; Xiao et al., 2017). The impact of each lncRNA may be determined by its ability to perform numerous tasks in the cell by interacting with proteins, DNA and RNA molecules.

To assist in understanding the pathogenesis of complex diseases, there have been efforts to infer potential associations between lncRNAs and diseases using lncRNA-protein interaction data (Chen and Yan, 2013; Li et al., 2014; Yang et al., 2014; Liu et al., 2017). Computational approaches have been developed to predict lncRNA-protein interactions, such as lncPro and RPI-Pred (Suresh et al., 2015; Shi et al., 2017; Xiao et al., 2017; Zheng et al., 2017). Several computation methods like LncRNADisease (Wang et al., 2016), GrwLDA (Gu et al., 2017), TPGLDA (Ding et al., 2018), and KATZLDA (Chen, 2015) uncover potential lncRNA-disease associations by integrating lncRNA functional similarities, lncRNA expression profiles, known lncRNA-disease associations, disease semantic similarities, and gene-disease associations. Studies seeking to predict non-coding RNAs in disease by constructing heterogenous networks with multiple types of biological interactions include ncPred, which uses resource transfer on a tripartite network (Alaimo et al., 2014); ComiRNet, which applies clustering to miRNA-gene regulatory networks (Pio et al., 2015); and LP-HCLUS, which integrates across interactions between lncRNAs, miRNAs, diseases, and genes (Barracchia et al., 2018). Most of these approaches use experimentally known lncRNA-disease associations as part of their input data and infer new lncRNA-disease associations.

An emergent concept postulates that a disease reflects the interplay of multiple biomolecules, and is rarely a straightforward consequence of an abnormality in a single gene encoding protein (Barabasi et al., 2011; Vidal et al., 2011; Sharma et al., 2015; Tasan et al., 2015; Sonawane et al., 2019). Given that each lncRNA may regulate multiple protein targets, and each protein may interact with multiple lncRNAs and with other proteins, it is crucial to integrate lncRNA-protein and protein–protein interactions in a heterogeneous network model to fully understand their dynamics at a molecular level. As the prediction of lncRNA disease associations are at a very early stage, known lncRNA-disease associations are limited. Here, we use the information flow-based method that exploits the connectivity structure among proteins and lncRNAs to predict novel lncRNA-disease associations. Diffusion-based methods are based on the notion that products of genes associated with diseases have a strong tendency to interact with each other in terms of the cumulative strength of paths that connect the corresponding proteins. These methods estimate the most redundant paths on the network, identifying the destination nodes (lncRNAs) that are most likely to be reached when starting from the seeds (disease proteins). When a node has a high score it means that the paths leading to it are highly redundant, which in turn implies that even if a portion of the network edges were missing due to incompleteness of data the results would be similar. This is in contrast with shortest-path-based methods that can instead be very sensitive to the removal of some critical links. We and others have previously proposed network diffusion approaches that model the information flow in molecular networks to localize the disease network neighborhood (Sharma et al., 2018), identify biomarkers in genome-wide studies (Qian et al., 2014), find significantly mutated pathways in cancer (Vandin et al., 2011), and prioritize disease genes (Navlakha and Kingsford, 2010). These studies successfully exploit the topology or structure of molecular interactions, called the interactome, even in incomplete space. With the growing availability of lncRNA-related interactome data, generalizing the guilt-by-association principle to predict lncRNA candidates might help in revealing the role of lncRNA in complex, interconnected disease mechanisms.

In this work, we propose **L**ncRNA rank**I**ng by Netw**O**rk Diffusio**N** (LION). LION is a network-diffusion method that integrates lncRNA-protein, protein–protein, and disease-protein networks to prioritize important lncRNAs for diseases. First, we construct a multi-level complex network (tripartite network) consisting of lncRNA-protein, protein–protein, and protein-disease associations. Next, we apply a random walk network diffusion algorithm. The random walk method exploits the local network neighborhood of diseases to measure the proximity of lncRNAs to the disease genes based on the probabilities of the connecting edges. It is possible to identify which lncRNA is connected to a given disease on the basis of the probability of reachability in the heterogeneous network. To evaluate LION, we utilize the available experimentally verified lncRNA disease associations (Chen et al., 2013) to demonstrate the performance of our method and compare with a similar method (TPGLDA) to demonstrate the performance advantages of our approach.

## Results

### Predicting the LncRNA-Disease Network

**FIGURE 1:**
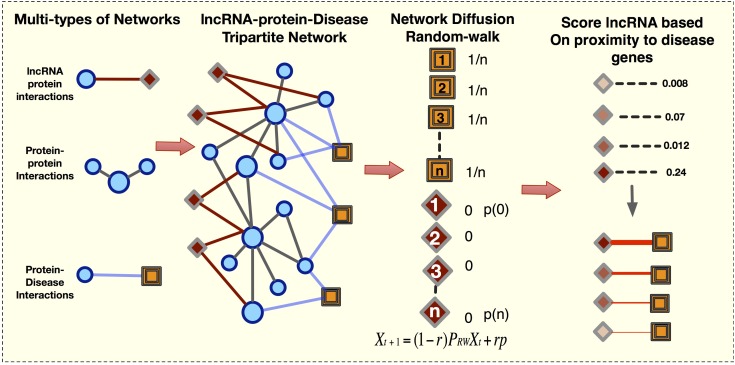
Framework to create the lncRNA-Disease-Network (LDN). We first construct the lncRNA-gene-disease tripartite network and next apply network diffusion method to rank lncRNA disease associations.

The majority of current methods (Chen and Yan, 2013; Liu et al., 2014; Sun et al., 2014; Yang et al., 2014; Lu C. et al., 2018) use the known lncRNA-disease interactions to compute the novel associations. Here, we predicted the lncRNA-disease network without *a priori* lncRNA-disease information. We first constructed a tripartite network from 28,488 protein-disease associations compiled from OMIM and GWAS databases, 141,296 protein–protein interactions, and 3,998 lncRNA-protein interactions (Figure 1). Next, we applied LION to prioritize lncRNAs, computing the probability of a random walker moving from a disease protein to a lncRNA. In the end, a final bipartite lncRNA-disease network was constructed from the predictions of 747 diseases. This lncRNA-disease network consisted of 304,868 weighted lncRNA-disease edges, where each link represents a predicted association between a disease and lncRNA that is proximal to its corresponding disease genes.

### Evaluating LION Predictions

We used the LncRNADisease experimental lncRNA-disease dataset to assess the predictive ability of LION, described in materials and methods. The dataset contains 372 lncRNAs, 245 diseases, and 1,101 lncRNA-disease associations. We calculated area under the receiver operating characteristic (ROC) curve to evaluate the predictive performance of our method. For the receiver operating characteristic curve, we plotted sensitivity and specificity at different thresholds, using the predicted lncRNA-disease edge weights as the thresholds. A predictor making random guesses would have an AUC of 0.5 and a predictor with perfect performance would have an AUC of 1. Typically, an AUC above 0.85 is considered good performance and an AUC above 0.95 is considered excellent performance.

We assessed the relative performance of LION by computing and analyzing three ROC curves: (1) LION method, (2) a current state-of-the-art method called TPGLDA, (3) a randomized network model as a negative control. TPGLDA uses known lncRNA-disease associations and known gene-disease associations, and makes predictions using a resource allocation algorithm that creates interaction profiles at each lncRNA. We generated the random network by starting with the lncRNA-disease network predicted with LION, and randomly shuffling node labels to create a random graph null model with the same connectivity structure, enabling comparison with LION’s predictions as a control.

**FIGURE 2:**
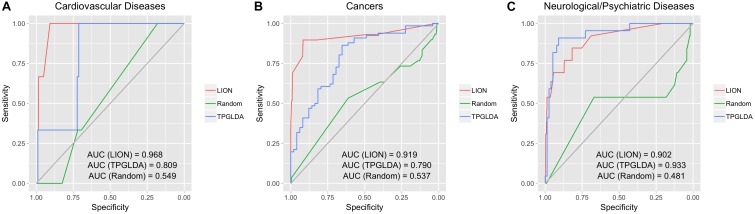
LION’s performance in predicting lncRNA-disease associations for three broad groups of diseases. For each, three receiver operating characteristic (ROC) curves are shown: (1) LION, (2) TPGLDA, a current state-of-the-art method for lncRNA-disease association prediction, (3) randomized network generated with node label shuffling as a negative control. Area under the ROC curve (AUC) values are listed for each ROC curve. **(A)** ROC plot for cardiovascular disease. **(B)** ROC plot for cancers. **(C)** ROC plot for neurological and psychiatric diseases.

To assess the overall performance of LION in predicting lncRNA-disease associations, we first applied LION to predict lncRNA-disease associations for three broad categories of diseases: cardiovascular diseases, cancers, and neurological diseases (Figure 2). LION yielded high performance for all three with AUCs all above 0.9. In contrast, the randomized network had a low AUC of approximately 0.5, which corresponds to a predictor making random guesses when the lncRNA-disease associations themselves are randomly assigned. The high AUCs above 0.9 with LION indicates our method is accurately predicting biologically relevant lncRNA-disease associations by inferring them from interactome and lncRNAome data. The AUC performance of TPGLDA was 0.809, 0.790, and 0.933 for cardiovascular disease, cancers, and neurological disease, respectively. Compared to TPGLDA, LION has improved performance in cardiovascular diseases and cancers, and comparable performance in neurological diseases. This confirms the ability of LION to make equivalent or higher accuracy predictions with respect to TPGLDA; in particular, LION does so without experimental lncRNA-disease data as an input.

**FIGURE 3:**
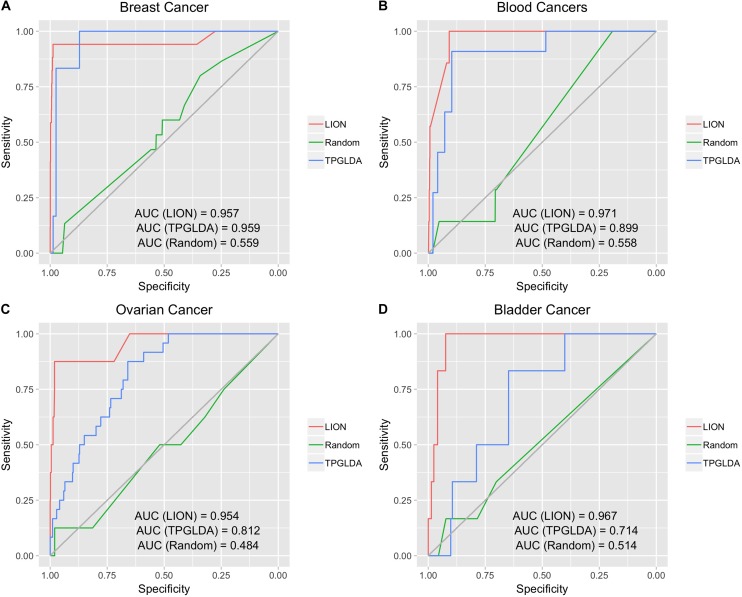
Performance in predicting lncRNA-disease associations for four individual cancers. LION outperformed the random network on all four cancers, and had higher or comparable performance than TPGLDA for all four. **(A)** ROC plot for breast cancer. **(B)** ROC plot for blood cancers. **(C)** ROC plot for ovarian cancer. **(D)** ROC plot for bladder cancer.

Having demonstrated high performance for three broad disease groups, we next evaluated LION on four individual cancers (Figure 3). The computed AUCs for LION were 0.957, 0.971, 0.954, and 0.967 for breast cancer, blood cancers, ovarian cancer, and bladder cancer, respectively. With AUCs exceeding 0.95, LION demonstrated excellent performance in predicting lncRNAs for individual cancers. Similar to the broad disease groups of Figure 2, the random network ROC curve for the cancers had much lower AUCs of around 0.5. When compared against the TPGLDA method, we see that LION has an improved or roughly equal performance to TPGLDA; AUCs for TPGLDA were 0.959, 0.899, 0.812, and 0.714 for breast cancer, blood cancers, ovarian cancers, and bladder cancers, respectively. For breast cancer and blood cancers, performance was roughly equal in both methods, while in the case of ovarian and bladder cancer, LION outperformed TPGLDA. These results further confirm the promise of LION to make accurate predictions from interactome and lncRNA-protein data, without prior information of lncRNA-disease associations.

We tested the statistical significance of differences in predictions made by LION, the randomized network, and TPGLDA methods using the Wilcox rank sum test. For each of the 7 diseases in Figure 2, 3, we ran two Wilcox tests: (1) comparing edge weights between LION and the randomized network, (2) comparing edge weights between LION and TPGLDA. We found statistically significant differences between LION’s predictions and the randomized predictions (*p* < 0.01), and between LION and TGPLDA (*p* < 0.01). The results of the Wilcox test confirm that LION’s better performance, seen in Figure 2, 3, arise from an improved ranking of disease-associated lncRNAs that are significantly different from either the TGPLDA rankings or from the randomized rankings.

### Top LncRNAs for Breast, Blood, Ovarian, and Bladder Cancer

We next conducted a qualitative evaluation of the top 50 predicted lncRNAs for four individual cancers to gain insight into their biological relevance to the disease. We identified biological roles of lncRNAs using the LncRNADisease database and experimental studies in the literature. For breast cancer, the top 50 predictions contained eight of the nine experimentally validated lncRNAs for breast cancer reported in LncRNADisease. The eight lncRNAs were MALAT1, XIST, HOTAIR, MEG3, GAS5, H19, CDKN2B-AS1, and PVT1. Each of these lncRNAs been validated by genetic profiling studies or *in vivo* and *in vitro* experimental studies, reported in the LncRNADisease database. For example, MALAT1 is a regulator of alternative splicing in breast cancer (Moran et al., 2012; Qi and Du, 2013; Jiang and Bikle, 2014); HOTAIR is overexpressed in a quarter of breast cancers (Gupta et al., 2010; Hung and Chang, 2010); the role of XIST in X chromosome inactivation is linked to BRCA1 tumors (Vincent-Salomon et al., 2007); MEG3 suppresses breast cancer through AKT (Zhang et al., 2017); GAS5 is downregulated in breast cancer and can induce apoptosis (Mourtada-Maarabouni et al., 2009). H19 is the direct target of a critical breast oncogene that triggers tumorigenesis (Barsyte-Lovejoy et al., 2006). CDKN2B-AS1 was identified through a GWAS study of breast cancer susceptibility loci (Turnbull et al., 2010). PVT1 was mechanistically linked to a common loci of breast cancer risk through its role as an apoptotic inhibitor (Guan et al., 2007). Apart from these eight, several additional predictions in the top 50 have been recently validated and reported in the literature, but not yet added to the LncRNADisease database. These lncRNAs include DANCR, NEAT1, RMST, and KCNQ1OT1 (Ke et al., 2016; Sha et al., 2017; Feng et al., 2018; Wang et al., 2018). One of the top ranked lncRNAs, HULC, has been linked to other forms of cancer but not to breast cancer. HULC decreases miRNA 15a, which in turn increases expression of p62, a critical cancer signaling protein; both miR15a and p62 have been linked to breast cancer (Patel et al., 2016; Xu et al., 2017). Given the biological roles of HULC’s two known targets in breast cancer pathways, HULC may be a potentially important lncRNA for breast cancer.

For bladder cancer, we found six experimentally validated lncRNAs in the LncRNADisease database, all of which were in the top 50 LION predictions. Of these, XIST, H19, MALAT1, MEG3 play a role in tumor proliferation, suppression, and metastasis of bladder cancer (Ariel et al., 2000; Ying et al., 2013; Martens-Uzunova et al., 2014; Wu et al., 2014). UCA and TUG1 are both known to promote cell proliferation and tumorigenesis in bladder carcinomas (Wang et al., 2012; Han et al., 2013). A top ranked lncRNA prediction, DBH-AS1 has not been linked to bladder cancer experimentally, but it regulates the miRNA-138-5p, a key inducer of bladder cancer carcinogenesis (Yang et al., 2016; Bao et al., 2018). Thus, DBH-AS1 may be a potential novel lncRNA target for bladder cancer.

A qualitative evaluation of ovarian cancer revealed the top 20 predictions by LION contained all four experimentally validated lncRNAs: MALAT1, H19, HOTAIR, and PVT1. Via the MAPK pathway, MALAT1 promotes ovarian cancer cell proliferation and migration (Zou et al., 2016). H19 has been investigated as a targeted therapy strategy for ovarian cancer and its inhibition suppresses tumor growth (Mizrahi et al., 2009). HOTAIR is a predictor of patient prognosis, including features such as tumor grade and survival (Qiu et al., 2014). Inhibition of PVT1 is linked to induction of an apoptotic response and proliferation inhibition of ovarian cancer cell lines [17908964]. The lncRNA TERC was highly ranked, but our literature search did not uncover a study linking TERC and ovarian cancer. A component of the telomerase enzyme complex, TERC is implicated in maintaining telomere length and therefore genetic susceptibility to aging related diseases, such as ovarian cancer (Grammatikakis et al., 2014). Our ranking and literature search on the biological role of TERC suggests it may be a new lncRNA that could be related to ovarian cancer.

The top 50 ranked lncRNAs for blood cancer contained 4 of the 11 experimentally validated lncRNAs. Polymorphisms in the gene encoding lncRNA CDKN2B-AS1 are associated with lymphoblastic leukemia (Iacobucci et al., 2011). A chromosomal translocation mutation implicated in B-cell lymphoma was linked to the GAS5 lncRNA; the mutation causes fusion of the GAS5 transcript to the BCL6 gene (Nakamura et al., 2008). Similarly, a common chromosome eight breakpoint mutation in Burkitt’s lymphoma was linked to the PVT1 locus in a mouse model, implicating PVT1 in disease tumorigenesis (Graham and Adams, 1986). In a genetic profiling study of acute myeloid leukemia patients, hypermethylation of the imprinted gene MEG3 was linked to significantly reduced survival (Benetatos et al., 2010). Similar to breast cancer, our literature search uncovered several high ranked lncRNAs that are linked to blood cancers but yet not been added to the LncRNADisease database, such as MIAT, MALAT1, XIST, CRNDE (Ellis et al., 2012; Yildirim et al., 2013; Sattari et al., 2016; Ahmadi et al., 2018). One of the top ranked lncRNAs, SNHG15, has not been experimentally linked to blood cancer; however, its regulation of the ubiquitin-proteasome system and its established oncogenic role in osteosarcoma suggest it may be a promising lncRNA for blood cancers (Ireland, 1986; Jiang et al., 2018).

### Case Study of Myocardial Infarction

As the role of lncRNAs in myocardial infarction has been well studied, we examined predictions for myocardial infarction as a case study to further validate our lncRNA-disease predictions. Myocardial infarction is one of the world’s leading causes of death (Benjamin et al., 2017). As shown in Table 1, all top five lncRNAs are not only validated in the experimental lncRNA-disease dataset, but also by experimental studies in the literature, providing further validation for our method. For example, clinical studies have found HOTAIR is downregulated in serum of patients and *in vivo* experiments revealed HOTAIR has a cardioprotective role (Gao et al., 2017; Lu W. et al., 2018). This indicates that HOTAIR is a promising candidate as a clinical biomarker for non-invasive diagnosis and potential therapeutic target for myocardial infarction.

**Table 1:** Top five lncRNA predictions by LION for Myocardial Infarction.

Rank	Disease	LncRNA	Validation (PMID)	Study description
1	Myocardial infarction (MI)	HOTAIR	29258067, 30468490	HOTAIR expression is decreased in serum of MI patients. Overexpression of HOTAIR prevents myocyte apoptosis
2	Myocardial infarction	PTCSC3	28982122	SNP in PTCSC3 is a genetic risk variant for CVD and MI in patients with autoimmune diseases
3	Myocardial infarction	GAS5	30099044, 29267258	GAS5 ameliorates cardiomyocyte apoptosis induced by MI, by down-regulating sem3a protein. GAS5 is downregulated in serum of patients with coronary artery disease, a risk factor for MI
4	Myocardial infarction	XIST	29226319	XIST is overexpressed in post myocardial cells. XIST promotes MI by regulating miR-130a-3p
5	Myocardial infarction	NEAT1	30924864	NEAT1 is suppressed in MI patients. NEAT1 knockout in a mouse model disrupted immune functions and caused myocardial inflammation


*All five have been validated by genetic profiling studies or experimental studies on cell lines and animal models. The PMID and a brief summary of each validating study are listed for each lncRNA.*

### Novel LncRNAs for Respiratory Diseases

Since LION does not rely on known, experimentally verified lncRNA-disease associations to make predictions, it is not restricted to only diseases for which experimental data is available. We applied LION to predict potential novel lncRNAs for respiratory diseases, where the role of lncRNAs is least explored. Respiratory diseases are a class of genetically complex diseases where the molecular and regulatory genomic underpinnings, and particularly the role of lncRNAs, are not well understood. In particular, we focused on respiratory tract infections (RTIs) and chronic obstructive pulmonary disease (COPD).

Lower RTIs are the most common infection and one of the leading causes of death in the United States by infection (File, 2000). COPD is the third leading cause of death in the United States, with about 15 million cases per year in the United States (Doney et al., 2014). Tables 2, 3 show the top five predictions for RTIs and COPD, respectively. None of the top predicted lncRNAs have been linked to RTIs. Of the lncRNAs predicted for COPD, only MEG3 has been associated with COPD in the literature. The unconfirmed lncRNAs present novel potential lncRNAs that could be further studied as regulatory drivers of the disease, clinical biomarkers, and therapeutic targets. MEG3, a top lncRNA for both COPD and RTIs, is differentially expressed in pulmonary fibrosis and in COPD (Tang et al., 2016; Gokey et al., 2018), indicating it may play a key role in the pathology of multiple respiratory diseases. IFNG-AS1, a top lncRNA for only RTIs but not COPD, has been linked to T helper cell responses (Peng et al., 2015), suggesting it may play a role in the immune response component of RTIs. RTIs, COPD, and myocardial infarctions all have HOTAIR in the top five predictions. This suggests HOTAIR’s roles in gene methylation and epigenetic differentiation may contribute to it being strongly implicated in many diseases caused by a combination of environmental and genetic factors.

**Table 2:** Top five lncRNA predictions by LION for respiratory tract infections.

Rank	Disease	LncRNA
1	Respiratory tract infections	DBH-AS1
2	Respiratory tract infections	MEG3
3	Respiratory tract infections	IFNG-AS1
4	Respiratory tract infections	HOTAIR
5	Respiratory tract infections	MALAT1


**Table 3:** Top five lncRNA predictions by LION for Chronic obstructive pulmonary disease (COPD).

Rank	Disease	LncRNA	PMID
1	Chronic obstructive pulmonary disease	H19	
2	Chronic obstructive pulmonary disease	XIST	
3	Chronic obstructive pulmonary disease	HOTAIR	
4	Chronic obstructive pulmonary disease	GAS5	
5	Chronic obstructive pulmonary disease	MEG3	27932875


## Discussion

Motivated by the success of network based methods in extrapolating information from the interactome (Navlakha and Kingsford, 2010; Vidal et al., 2011; Menche et al., 2015; Sharma et al., 2018), we develop a lncRNA-gene-disease tripartite graph and collapse it into a weighted lncRNA-disease bipartite graph using a random walk. The powerful predictive ability and applicability of LION for virtually any disease arises from its unique method of multi-layer network construction and ranking with a network diffusion algorithm. Accumulating evidence suggests the importance of lncRNA’s role in various biological processes and in predicting novel lncRNAs for diseases has significant medical and biological implications. Most of the current methods infer potential lncRNA disease associations based on existing knowledge of lncRNA-disease relationships (Chen and Yan, 2013; Chen, 2015; Gu et al., 2017; Ding et al., 2018). In contrast to current state-of-the-art methods (Chen et al., 2017), we predicted lncRNA-disease associations by using the topology of a heterogeneous network comprising lncRNA-proteins, protein–protein, and protein-disease interactions. Moreover, LION is not restricted to predicting lncRNAs for diseases with known lncRNAs, it can make predictions for any disease by using their known disease proteins. To our knowledge, this approach is the first of its kind to make accurate predictions from protein interactome and lncRNA-protein data, without requiring known lncRNA-disease associations.

Making lncRNA-disease predictions without *a priori* information does not impact the performance of LION. When compared against experimental lncRNA-disease associations, LION accurately predicted lncRNAs for both broad disease categories (cancers, cardiovascular diseases, and neurological/psychiatric diseases) and individual cancers (breast, blood, ovarian, and bladder), with AUCs all over 0.85. In contrast, the negative control, a lncRNA-disease network randomized with node label shuffling, had AUCs of only 0.5. In comparison to the current state-of-the-art method for lncRNA-disease prediction, TPGLDA, LION had improved or equal performance. LION had improved performance for cardiovascular diseases, cancers, ovarian cancer, and bladder cancer. LION performed equally well as TPGLDA on neurological/psychiatric diseases, breast cancer, and blood cancers. LION has potential value for predicting novel lncRNAs, demonstrated through analysis of case studies in COPD and respiratory tract infections. LION has potential applications in biomedical research to shed light on molecular underpinnings of disease, prioritize putative therapeutic targets and biomarkers, and elucidate disease-disease relationships.

Despite its promise, the limitations that influence the prediction of LION include the literature biases in the protein-disease, lncRNA-proteins, and protein–protein interactions datasets. In particular, as lncRNA-protein associations are likely quite incomplete owing to the recent discovery of lncRNAs, the lncRNA-protein dataset may be skewed toward a few well studied lncRNAs. Furthermore, the missing links could bias the predictions toward the well-studied lncRNAs, such as HOTAIR, which we found in the top five predictions for myocardial infarction, COPD, and respiratory tract infections. A second limitation is the unweighted and incomplete GWAS and OMIM gene-disease datasets used to build the tripartite network; LION would be improved by weights that enable distinguishing crucial disease genes, such as through using differential gene expression data. We plan to include more complete interaction datasets to improve the accuracy of prediction in the future.

## Materials and Methods

### Data Sources and Construction of Multi-Level Complex Network

To obtain genome-wide lncRNA and protein-coding gene associations, we combine three sources:

#### LncRNA-Protein Interaction Data

We downloaded known lncRNA-protein interaction datasets from the following databases. (i) lncRInter, a reliable and high quality lncRNA interaction database containing experimentally validated data whose lncRNA interaction datasets are extracted from peer-reviewed publications (Liu et al., 2017). (ii) NPInter v3.0 contains experimentally verified interactions between non-coding RNAs, especially lncRNAs, and other molecules (proteins, mRNAs, genomic DNAs) (Hao et al., 2016). (iii) EVLncRNAs, a high-quality and integrated database that manually curates all types of experimentally validated lncRNAs (Supplementary Table S1; Zhou et al., 2018).

#### RNA-Binding Protein (RBP)-LncRNA Interactions

By analyzing millions of RBP binding sites from 117 CLIP-Seq datasets generated by 50 independent studies, starBase V2.0 has identified 22,735 RBP–lncRNA regulatory relationships (Supplementary Table S1; Li et al., 2014).

#### Protein–Protein Interactions Network

We combine several sources of protein interactions: (i) regulatory interactions derived from transcription factors binding to regulatory elements; (ii) binary interactions from several yeast two-hybrid high-throughput and literature-curated datasets; (iii) literature-curated interactions derived mostly from low-throughput experiments; (iv) metabolic enzyme-coupled interactions; (v) protein complexes; (vi) kinase-substrate pairs; and (vii) signaling interactions. The union of all interactions from (i) to (vii) yields a network of 15,949 proteins that are interconnected by 217,140 interactions (Supplementary Table S2; Menche et al., 2015).

#### Disease-Protein Interactions Network

A total of 28,488 associations between protein-coding genes and diseases in the OMIM and GWAS were downloaded from DisGeNET (Supplementary Table S3; Pinero et al., 2017).

### Network Diffusion Algorithm to Infer Key Candidate LncRNAs

To predict and rank disease associated lncRNAs, we first constructed a tripartite lncRNA-protein-disease network from the datasets described in section “Data Sources and Construction of Multi-Level Complex Network” (Figure 1).

Next, we utilized a random walk with restart to rank lncRNAs for each disease using network based proximity between a lncRNA and a disease’s known proteins (Kohler et al., 2008). For this, we constructed a subnetwork of the tripartite network, comprised of all the disease genes and their nearest neighbors, as well as the lncRNAs regulating the proteins. We performed this step to localize the lncRNA predictions to each disease network neighborhood in the interactome. Indeed, based on the local impact hypothesis, molecular entities involved in similar diseases have an increased tendency to interact with each other and to localize in a specific network neighborhood (Sharma et al., 2015).

For example, with myocardial infarction (MI), we created a subnetwork with (1) 1228 protein–protein interactions between the 27 known MI genes and their nearest neighbors, and (2) 2,175 lncRNA-protein interactions involving 1235 lncRNAs. The MI seed genes are included in Supplementary Table S4 and the adjacency matrix representing the MI subnetwork is in Supplementary Table S5.

We then executed a random walk process to predict the lncRNA associated to the 27 known MI genes. The basic idea of LION is a walker starting from a single or group of disease genes and visiting other genes and lncRNAs (nodes) in the multi-level network by taking a series of random walking steps. On every moving step, the walker moves from its current node to the neighboring nodes and therefore a distribution value is calculated for every node in the network, which denotes the probability that a walker is at a given node at the current step. At each step, the walker has a probability *r* = 0.5 to be relocated on the starting genes. The probability distribution at step *k* + 1 is described by the iterative form:

$$p^{k+1}=(1-r)Wp^{k}+rp^{0}$$

where, *p^k^* is a vector where the *i*-th element is the probability of being at node *i* at step *k. p*^0^ is the uniform distribution over all starting disease genes. *W* is the column normalized graph adjacency matrix. By iterating the process until convergence (|| *p*^k+1^ − *p*^k^ || _L1_ < 10^-6^) we obtained the steady state probability *p*^∞^, and 1235 lncRNAs were ranked according to their values in *p*^∞^. Pseudocode for the network diffusion algorithm is included in the Supplementary Information.

This process was repeated for all of the 747 diseases for which a non-bipartite and connected subnetwork could be constructed. These conditions are required for the random walk probabilities to converge to a unique limiting probability distribution.

The final predicted lncRNA-disease network contains 304,868 weighted lncRNA-disease associations between 747 diseases and 1,346 lncRNAs. Each association represents a predicted link between a disease and a lncRNA proximal to the disease’s genes. The number of lncRNAs ranked for each disease has a median of 156 and the number of diseases targeted by each lncRNA has a median of 195. Supplementary Table S6 contains the predicted lncRNA-disease associations for the 4 types of cancer (breast, bladder, blood, and ovarian) and 3 cardiovascular diseases (myocardial infarction, respiratory tract infections, and chronic obstructive pulmonary disease), analyzed in the section “Results.”

### Validation of LION Using LncRNADisease Experimental Dataset

To validate the lncRNA-disease predictions by LION, we use the LncRNADisease dataset (Chen et al., 2013), a manually curated database of 1,101 experimentally validated lncRNA-disease associations between 245 diseases and 372 lncRNAs. Using the LncRNADisease experimental dataset as ground truths and predicted lncRNA-disease edge weights from LION, we create receiver operating characteristic (ROC) curves and compute area under the ROC curve (AUC). We created ROC curves first for broad disease categories – cancers, cardiovascular diseases, and neurological/psychiatric diseases – and next for specific diseases – breast, bladder, ovarian, and blood cancers. As a negative control, we created a random graph null model by shuffling node labels on the bipartite lncRNA-disease network. To assess if LION is performing as well as current methods, we compare with the TPGLDA method (Ding et al., 2018). Using the same experimental LncRNADisease dataset, we also create ROC curves for both TPGLDA and the randomized network.

## Author Contributions

AS and MS conceived the idea of the study. SW co-supervised the analyses. MS and EM performed the computational and statistical analyses. All authors contributed to the interpretation of the results and in writing the manuscript, read, and approved the final manuscript.

## Conflict of Interest Statement

The authors declare that the research was conducted in the absence of any commercial or financial relationships that could be construed as a potential conflict of interest.

## Supplementary Material

TABLE S1 Dataset of lncRNA-protein interactions. Contains 391,791 interactions from lncRNAInter, NPInter, EVLncRNAs and starBas between 1,779 lncRNAs and 14,951 proteins.

TABLE S2 Dataset of protein-protein interactions. Contains 217,160 interactions between 15,961 proteins.

TABLE S3 Complete dataset of disease-gene associations downloaded from DisGeNET. Contains 28,488 disease-gene associations between 4,454 diseases and 6,464 genes.

TABLE S4 Set of 27 known disease genes for myocardial infarction for use in seeding a random walk.

TABLE S5 Adjacency matrix representing an example subnetwork for myocardial infarction.

TABLE S6 Predicted lncRNA-disease associations for breast cancer, blood cancer, bladder cancer, ovarian cancer, myocardial infarction, respiratory tract infections, and chronic obstructive pulmonary disease.

DATA SHEET S1 Pseudocode for LION algorithm.
