# Supplementary material for: Network Diffusion Approach to Predict LncRNA Disease Associations Using Multi-Type Biological Networks: LION
Source: Front Physiol. 2019 Jul 16;10:888. doi: 10.3389/fphys.2019.00888 (PMC6646690; doi:10.3389/fphys.2019.00888)
Supplement: DATA SHEET S1 — Pseudocode for LION algorithm. [file Data_Sheet_1.pdf]

LION(*D, PPI, LP*)
input:
D: set of known disease proteins
PPI: protein-protein interaction network
LPI: lncRNA-protein interaction network
output:
L: the set of ranked candidate lncRNAs

PPI_sub ← subgraph of *PPI*, all edges involving a protein in *D*
LPI_sub ← subgraph of *LPI*, all edges involving a protein in *N*

S ← disease subnetwork, the union of *PPI_sub* and *LPI_sub*
N ← number of nodes of *S*

p^0 ← vector with size *N*, initialized to all zeros // probability vector for step 0
for each protein *i* **in** *D* **do**
p_i^0 ← 1 / (number of elements in D)
end for
$p^k \leftarrow p^0$ // probability vector for step *k*
r ← 0.5 // restart probability
W ← adjacency matrix of *S*, column normalized
$p^{k+1} \leftarrow (1 - r) * (W \bullet p^k) + r * p^0$ // probability vector for step *k+1*
while $\| p^{k+1} - p^k \|_{L1} > 10^{-6}$ **do** // main iteration
$p^k \leftarrow p^{k+1}$
$p^{k+1} \leftarrow (1 - r) * (W \bullet p^k) + r * p^0$
end while
L ← subset of p^{k+1} representing all lncRNA nodes in *S*
return *L*
